# Recyclable carbon fibre composites enabled by cystine containing epoxy matrices†

**DOI:** 10.1039/c9ra06409e

**Published:** 2019-10-02

**Authors:** Martin L. Henriksen, Jakob E. Friis, Astrid Voss, Mogens Hinge

**Affiliations:** Plastic and Polymer Engineering, Department of Engineering, Aarhus University Aabogade 40, 8200 Aarhus N Denmark hinge@eng.au.dk

## Abstract

Recyclable composites are of industrial relevance and benefits the environment, which initiates research towards more sustainable solutions. In this study, a commercial epoxy thermoset, modified by a bio-based additive is used as an infusion resin making recyclable carbon fibre composites. The matrix fractionation process was investigated and optimized with respect to additive & solvent concentration, and temperature. Fully cured carbon reinforced composites were dismantled under the optimum condition and after drying, reinfused, and cured into a new composite, repeated three times on the same carbon fibre material. A decrease in fibre volume fraction and composite performance was found as a number of recyclings were performed. Finally, it was demonstrated that the recycled carbon fibre ply could be reshaped, infused, and cured and thus be applied in new components.

## Abstract 2

Carbon fiber composites are recyclable under mild conditions by using a bio-based additive in an epoxy system. Optimal conditions are investigated and even low additive amounts render the composite recyclable proven by multiple dismantling cycles.

## Graphical abstract

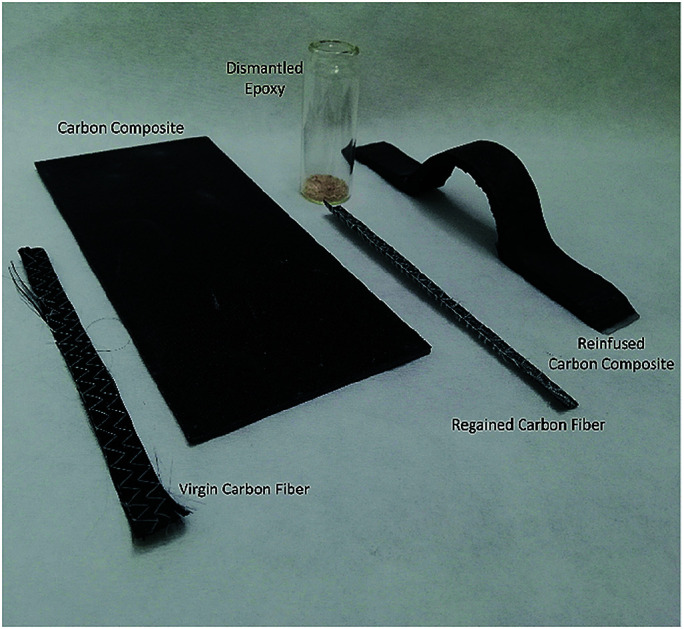

## Introduction

High-performance composite materials, like glass and carbon fibres embedded in a thermoset (polyurethane, polyester or epoxies) are gaining industrial interest^1^ mainly due to their properties as lightweight, high strength, and corrosive resistant materials. This interest is especially found within aerospace, transportation, and construction, exemplified by the Airbus A350 XWB, which has increased from 2–5% to 53% carbon composite in recent years.^2^ The main drawback is the lack of recyclability and/or reusability of cross-linked thermoset composites. The current solutions for large volume recyclability is solvolysis^3^ and incineration.^4^ These processes are industrialized^5^ and the recycled products are short discontinuous fibres. Although short fibres have many applications, these recycling processes are downgrading of the continuous fibres. Recycling composites while preserving the long fibres has initiated research towards chemical degradation of the matrix. Chemical matrix degradation has been studied *via* alkoxyamines,^6^ bond exchange reactions with ethylene glycol,^7^ urea bonds,^8^ acetals,^9^ carbonate,^10^ disulfide,^11^ esters^12^ and triazinane.^13^ Some of which are sensitive to water or UV and are therefore only applicable in protected environments (indoor, under a roof, *etc.*). Potential trigger solutions have not been industrialized either due to expensive and/or hazardous triggers or due to the degradation products. A recent study has demonstrated that the addition of 0.97 wt% of l-cystine to a commercial epoxy system rendered a matrix that could be fractionated in glacial acetic acid at 70 °C.^14^ Although promising results, the fractionation time is long and fibre recyclability is yet to be demonstrated.

This study demonstrates the recycling potential of carbon composites by addition of l-cystine to a commercial epoxy system and as a proof-of-concept the recycled carbon fibre fabrics are reinfused, and reshaped into new elements. Curing of clear cast epoxy matrices and the composites are monitored by infrared spectroscopy (FTIR) and differential scanning calorimetry (DSC). A time optimization of the matrix fractionation process is performed in relation to solvent mixture, l-cystine content, and temperature. Composites are infused *via* vacuum assisted resin transfer molding (VARTM) and the infusions are monitored by image analysis. The different resins' viscosities are determined by shear rheometry. Matrix, fibre, and void volume fraction (MVF, FVF, and VVF) are determined both by thermo-gravimetrical analysis (TGA) and by furnace burnout. Mechanical properties, morphology, and composite dismantling are investigated by three-point beam bending, optical microscopy, and scanning electron microscopy (SEM), respectively.

## Experimental

### General

**Scheme 1:**
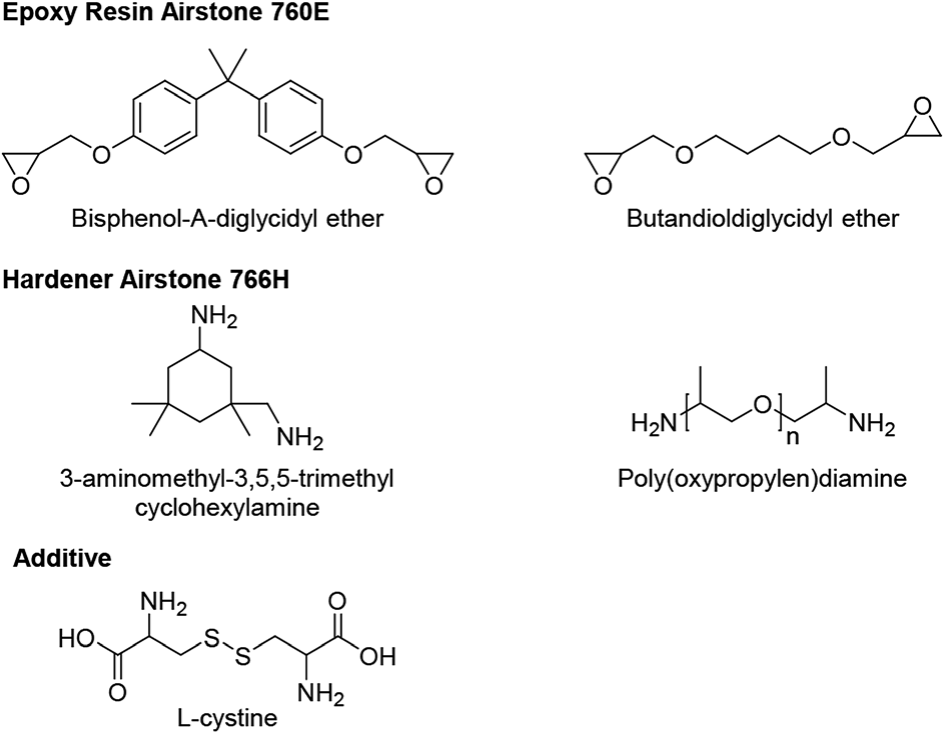
The epoxy resin, hardener and additive applied.

All materials are used as received, unless otherwise stated. Epoxy resin (760E, Airstone 760E) and hardener (766H, Airstone 760H) (Olin Corp., Germany). l-cystine (98.5%), methanol (HPLC, 99.9%), hydrochloric acid (37 wt%) all from Sigma-Aldrich. Glacial acetic acid (100%, Merck KGaA). Uni-directional carbon fabrics (333 GSM, Zoltek PX35, Zoltek Corp) (Scheme 1).

### Preparing and curing of standard and modified epoxy matrices

**Scheme 2:**
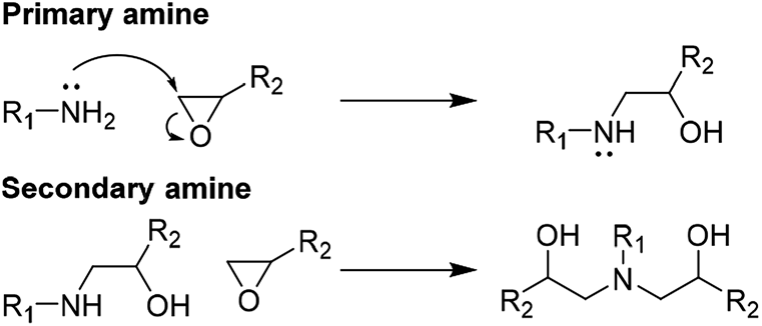
Curing reactions of the amine based epoxy resin.

The standard epoxy matrix is made by mixing 760E (100 g) with 766H (32.0 g), degassed for 20 min under vacuum, poured into preheated silicone moulds (50 °C) and cured at 50 °C for 1 h and 80 °C for 3 h (Scheme 2).

l-Cystine (5.12 g) and methanol (30 mL) is added to a 250 mL round-bottom flask and hydrochloric acid (10 mL) is added dropwise at ambient temperature till l-cystine starts swelling due to breakage of the strong hydrogen bond between the carboxylic acid and the amine groups.^14^ The swollen l-cystine is isolated by drying at 60 °C under vacuum. Dried l-cystine powder (5.12 g) is added to 760E (100 g) and reacted for 20 h at 100 °C assisted by ultrasound, generating a modified epoxy stock solution. This stock solution is diluted with 760E to obtain the l-cystine concentrations in Table 1. Hardener substitution is based on molar equivalent active sites and CYS_20_ denotes a 20 mol% l-cystine hardener substitution as described by ESI eqn (S1).†^14^ 766H is added, mixed, degassed and cured at 50 °C for 1 h and subsequently 80 °C for 3 h.^14^

**Table 1:** Concentrations for the applied epoxy matrices

ID	CYS_0_	CYS_2.5_	CYS_5_	CYS_10_	CYS_20_
760E [g]	100	100	100	100	100
766H [g]	32.0	31.2	30.4	28.8	25.6
l-cystine [g]	0.00	0.64	1.28	2.56	5.12

### Optimized dismantle time of epoxy matrices with respect to acetic acid concentration

2.0–2.2 g (4 mm *×* 10 mm *×* 50 mm) CYS_5_ samples are placed in an empty snap cap glass (25 mL, Thomas Scientific), which is filled to the rim with an aqueous (demineralized water) acetic acid at a mole fraction (*x*_AA_) of 0.07, 0.17, 0.24, 0.32, 0.42, 0.56, 0.64, 0.74, or 1.00 and sealed with a cap. A needle (∅ 1.2 mm by 40 mm, Sterican, B. Braun, DK) is pierced into the lid for pressure equalization and the vial is placed in a preheated oven at 70 °C (Steva, Elgiganten, DK), which is thermostatic controlled by a PID regulator (Sestos, D1S-VR-220, HK, CN). A camera (Digimicro, 2 MP, 10X - 200X, HooToo, GER) is placed in front of the oven and images are acquired every minute. Images are analyzed in ImageJ (v. 1.51r) employing a custom made macro (see ESI†), which identifies the sediment height based on contrast. Conversion is calculated as the percentage of the final sediment height. Conversion as a function of time is plotted and the fractionation rate is calculated as the slope in the range from 25% to 75% conversion and the dismantle time is then calculated as the extrapolated time from 0% to 100% conversion. The induction period is given as the time between the experiments is started and the extrapolated fractionation rate to 0%. The total dismantle time are given as the sum of the induction period and the dismantling time.

### Optimized dismantle rate of epoxy matrices with respect to temperature and l-cystine concentration

2.0–2.2 g (4 mm *×* 10 mm *×* 50 mm) CYS_2.5_, CYS_5_, and CYS_10_ samples are placed in an empty snap cap glass (25 mL, Thomas Scientific), which is filled to the rim with 0.64 mole fraction aqueous acetic acid and sealed with a cap. A needle (∅ 1.2 mm by 40 mm, Sterican, B. Braun, DK) is pierced into the lid for pressure equalization and the vial is placed in a preheated oven at 50, 70, 90, and 110 °C (Steva, Elgiganten, DK), setup and imaged as previously described. The calculated total dismantle time is divided by the mass of the test specimen and is given as the dismantle rate in mg min^−1^.

### Composite manufacturing and flow profile

Six layers of uni-directional carbon fabrics are infused with CYS_5_ applying the Vacuum Assisted Resin Transfer Moulding (VARTM) setup and method described elsewhere.^18^ The infusion table is preheated to 30 °C prior to infusion and the same curing profile as previous described is used for curing the panels. Images are acquired every second during the infusion and the flow profile is calculated using ImageJ (v. 1.51r). The slope of time (*t*) *versus* square length (*L*^2^) is determined and together with the integrated one dimensional Darcy's Law^19^ (eqn (1)) a permeability constant over the porosity is calculated:1
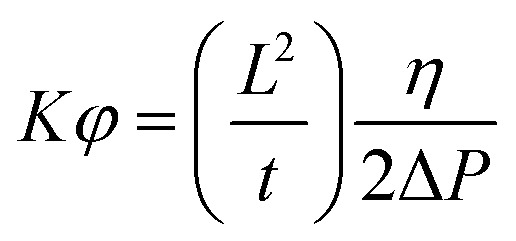
where, *K* is the intrinsic permeability constant [m^2^], *φ* is the porosity, *η* is the viscosity of the resin [Pa s], and Δ*P* is the pressure difference [Pa].

### Carbon composite recycling

A CYS_5_ carbon composite (2.4 mm *×* 220 mm *×* 150 mm) is submersed in an acetic acid solution (1 L, *x*_AA_ = 0.64) at 70 °C for ≈18 hours in a closed container. Hereafter, the solvent and matrix pulp are removed and the fabrics are left for drying for 24 hours at 70 °C. When dried, fabrics are gently brushed to remove loose residual epoxy pulp on the surface of the carbon fibre ply. The ply is then reinfused with CYS_5_ and cured as previous described into a recycled composite. The process of dismantling–drying–infusion–curing is repeated three times (denoted 2^nd^, 3^rd^, and 4^th^ infusion, respectively). For each cycle two 10 mm wide test specimens are cut in the 0° direction.

### Volume fractions

MVF, FVF, and VVF (eqn (2)) for the composites and recycled fibres are determined in two ways. (I) Samples of 20–50 mg are pyrolysed (N_2_ atmosphere) in a TGA (TG 209 F1 Libra, Netzsch) in the temperature range from 35 °C to 800 °C, 10 K min^−1^. (II) 10 g samples are burned in a furnace at 500 °C for six hours in normal lab atmosphere; weighted before and after the burnout.2
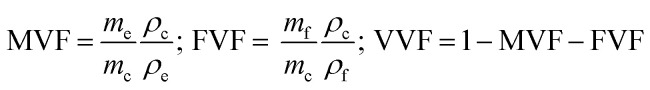
where *m*_e_ is lost epoxy matrix mass and *m*_f_ is remaining fibre mass. Epoxy matrix and fibre densities are *ρ*_e_ = 1.17 g mL^−1^ and *ρ*_f_ = 1.81 g mL^−1^, respectively. The composite density (*ρ*_c_) is found *via* Archimedes' principle (eqn (3)).3
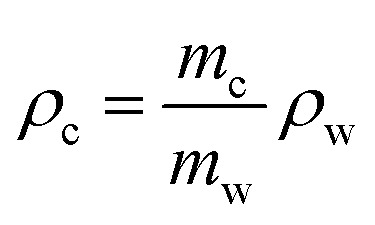
where the mass of the composite sample (*m*_c_) and the mass of the displaced water (*m*_w_), when the composite is submerged in demineralized water (*ρ*_w_ = 0.998 g cm^−3^ at 20 °C), is weighed on laboratory scale (Sartorius) at ambient temperature.

### Reshaped composite

A CYS_5_ carbon composite (2.6 mm *×* 100 mm *×* 300 mm) is prepared, dismantled and dried as previous described. The recycled ply is placed over a curved metal element in order to demonstrate both convex and concave reshaping. The stack is reinfused with CYS_5_ and subsequently cured as previous described.

### Average fibre diameter

Field emission scanning electron microscope, FEI NOVA nanoSEM 600, equipped with a low vacuum detector is applied in obtaining the SEM images. Images are measured at 60 Pa H_2_O, spot size 3, and 5 kV acceleration at 2k, 3k, or 8k magnification with a raster sampling time of 12 μs. Samples where mounted with carbon stickers. SEM images are post processed in ImageJ (v. 1.51r) to measure the thickness of the carbon fibres. Measurements (14) are performed on the virgin carbon fibres giving the average fibre diameter and its standard deviation.

### Optical granulometry

Particles from the fractionated matrices (pulp) are dried and spread on a black background with a ruler. High contrast images are acquired for each pulp. Images are calibrated according to the ruler, and the contrast adjusted to have black particles on white background, subsequently the particle area and diameter is calculated in ImageJ (v. 1.51r). After despeckling, calculations of average diameters and standard deviations are done in Excel.

### Infrared spectroscopy

Nicolet FTIR Spectrometer (iS5, Thermo Fisher Scientific, USA) equipped with an attenuated total reflectance (ATR) ZnSe crystal (iD7, Thermo Fisher Scientific, USA) controlled with OMNIC (Thermo Fisher Scientific, USA) acquired the infrared spectra. Cured samples were mounted firmly (with a torque wrench) and epoxy resin and hardener samples were applied dropwise onto the ATR crystal. Background (*n* = 32) is collected prior to sampling and subtracted the sample spectra (*n* = 200). Spectra post-processing: correction for wavelength-dependent penetration depth, baseline, and normalized by dividing the spectra with the peak intensity for the band at 828 cm^−1^ prior to analysis.

### Differential scanning calorimetry

A DSC 8000 (PerkinElmer) controlled by Pyris (PerkinElmer, v. 11.1.0.0488) is employed with constant nitrogen purging. Thermal program is: (I) hold for 0.5 min at 5.00 °C; (II) heat: 5.00 to 180.00 °C, 10.00 °C min^−1^; (III) cool: 180.00 to 5.00 °C, 40.00 °C min^−1^; (IV) heat: 5.00 to 180.00 °C, 10.00 °C min^−1^. (V) cool: 180.00 to 5.00 °C, 40.00 °C min^−1^. For all samples, the glass-transition temperatures (*T*_g_) is determined as inflection point after thermal normalization on the second heat scan (scan IV) by differential peak method.

### Rheology

The viscosity experiments are performed on a Discovery HR-2 (TA instruments) mounted with a 40 mm parallel plate (Peltier plate steel), controlled stress, run at an angular speed of 10.0 rad s^−1^, and at 30 °C. First, 760E, modified epoxy resin and 766H are mixed and run for 500 s, where after the initial viscosity is calculated as the average viscosity over all 500 s. Next, a fresh mixture is run for more than 4.5 hour, the pot life is taken as the time it takes to reach 1.0 Pa s.

### Flexural properties

Flexural modulus is determined *via* three-point-bending according to ISO 178 on a Z0.5 (ZwickRoll, Germany) with a support to support span of 64 mm. A preload of 0.1 MPa is applied and flexural modulus speed 2 mm min^−1^. Two composites samples (10 mm wide, 150 mm long, and thickness given in Table 3) are tested three times each and are run with a 500 N load cell and stopped at 200 N, whereas two virgin and recycled carbon fabrics are run three times each with a 10 N load cell and stopped at 5 N load. Flexural moduli are calculated from 0.05% strain to 0.25% strain following ISO 178.

### Cross sectional pictures

Carbon composites are cut in 1.0 cm wide panels using a multicutter (Fein MultiTalent) equipped with a diamond blade perpendicular to the fibre direction. The edge of the panels were polished using sanding paper (P120-P150-P320-P500-P1000-P1500) to obtain a smooth surface. Cross-sectional pictures were taking of the carbon composites using a digital single-lens reflex camera (Canon EOS 700D) equipped with a macroscopic lens (Canon MP-E 65 mm) mounted on a sledge. The polished carbon composites were mounted on an adjustable stage with background illumination.

## Results

### Curing analysis

**Fig. 1:**
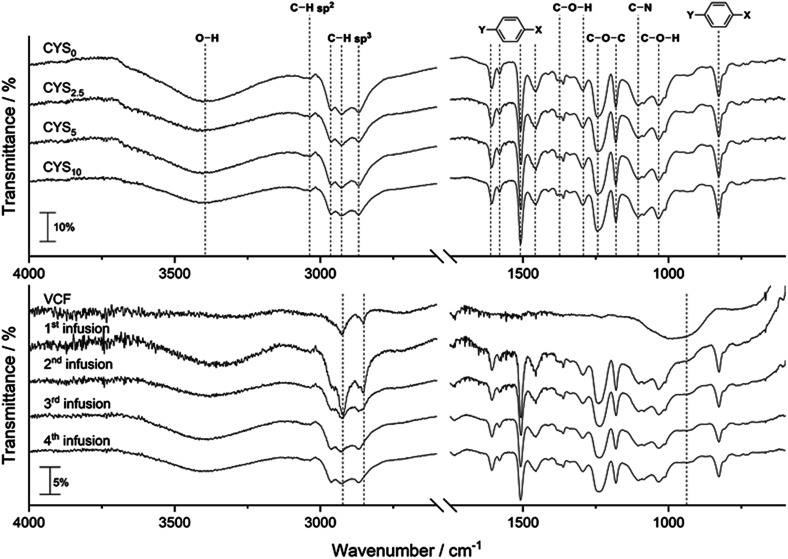
FTIR spectra of the pure epoxy matrices (top) with peak assignments and spectra of the VCF together with the infused composites (bottom). The dashed lines indicate the VCF bands at 960 cm^−1^, 2852 cm^−1^, and 2932 cm^−1^. All spectra except VCF are normalized by the peak found at 828 cm^−1^.

FTIR of cured clear cast epoxy matrices, virgin carbon fibre (VCF), and carbon composites are given in Fig. 1.

FTIR bands in Fig. 1 (top) for the cured matrices are assigned as: 3401 cm^−1^ O–H; 3034 cm^−1^ aromatic C–H sp^2^; 2962 cm^−1^, 2927 cm^−1^, 2868 cm^−1^ C–H sp^3^; 1606 cm^−1^, 1580 cm^−1^, 1508 cm^−1^, 1455 cm^−1^*para* aromatic C=C; 1362 cm^−1^ <svg xmlns="http://www.w3.org/2000/svg" version="1.0" width="10.400000pt" height="16.000000pt" viewBox="0 0 10.400000 16.000000" preserveAspectRatio="xMidYMid meet"><metadata> Created by potrace 1.16, written by Peter Selinger 2001-2019 </metadata><g transform="translate(1.000000,15.000000) scale(0.011667,-0.011667)" fill="currentColor" stroke="none"><path d="M80 1160 l0 -40 40 0 40 0 0 -40 0 -40 40 0 40 0 0 -40 0 -40 40 0 40 0 0 -40 0 -40 40 0 40 0 0 -40 0 -40 40 0 40 0 0 -40 0 -40 40 0 40 0 0 -40 0 -40 40 0 40 0 0 80 0 80 -40 0 -40 0 0 40 0 40 -40 0 -40 0 0 40 0 40 -40 0 -40 0 0 40 0 40 -40 0 -40 0 0 40 0 40 -40 0 -40 0 0 40 0 40 -80 0 -80 0 0 -40z M560 520 l0 -40 -40 0 -40 0 0 -40 0 -40 -40 0 -40 0 0 -40 0 -40 -40 0 -40 0 0 -40 0 -40 -40 0 -40 0 0 -40 0 -40 -40 0 -40 0 0 -40 0 -40 -40 0 -40 0 0 -40 0 -40 80 0 80 0 0 40 0 40 40 0 40 0 0 40 0 40 40 0 40 0 0 40 0 40 40 0 40 0 0 40 0 40 40 0 40 0 0 40 0 40 40 0 40 0 0 80 0 80 -40 0 -40 0 0 -40z"/></g></svg> C–H; 1362 cm^−1^, 1295 cm^−1^, 1035 cm^−1^, 828 cm^−1^ C–OH (sec. alcohol); 1245 cm^−1^, 1181 cm^−1^ C–O–C; 1105 cm^−1^ C–N amine; 828 cm^−1^*para* aromatic ring out-of-plane bending.^15^ The VCF (Fig. 1, bottom) shows a large signal-to-noise ratio and three bands at 960 cm^−1^ (broad), 2852 cm^−1^, and 2932 cm^−1^ are detected (indicated by dashed lines). Further, the epoxy matrix peaks in the composite are in accordance with the matrix bands found. It is seen that the noise and carbon fibre band intensity are decreasing as the number of infusions (*i.e.* the number of recycling) increases. FTIR of the recycled fibres prior to infusion can be found in ESI Fig. S1.† DSC showed a *T*_g_ of ≈78 °C with no post curing peak for all clear casts, composites, and recycled fibres (ESI Fig. S2–S7†), thus indicating all epoxy matrices are fully cured.

### Impact of acetic acid concentration on matrix fractionation

**Fig. 2:**
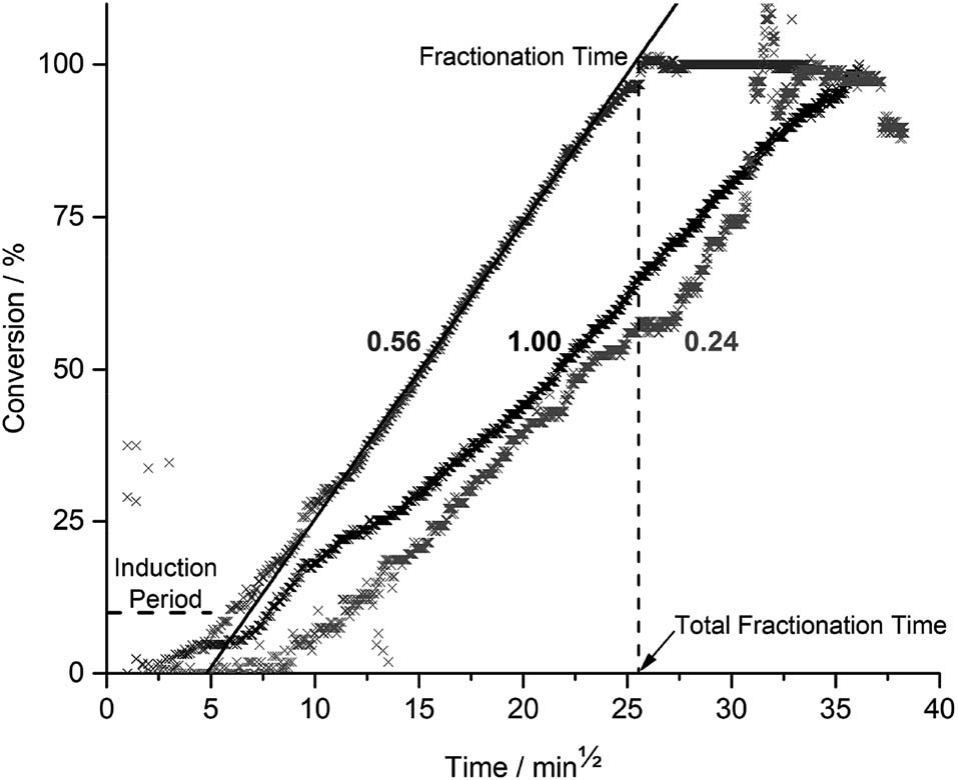
Clear cast CYS_5_ in 0.24 (light grey), 0.56 (dark grey), and 1.00 (black) *x*_AA_ at 70 °C as a function of square root time. The horizontal dashed line indicates the induction period, the solid line fitted from 25% to 75% conversion is used to calculate the fractionation rate, and the vertical dashed line indicates the total fractionation time.

During fractionation, cracking noises were observed as fragments detached and fell from the epoxy matrix. Fractionation as a function of time and acetic acid concentration (*x*_AA_) is given in Fig. 2.

**Fig. 3:**
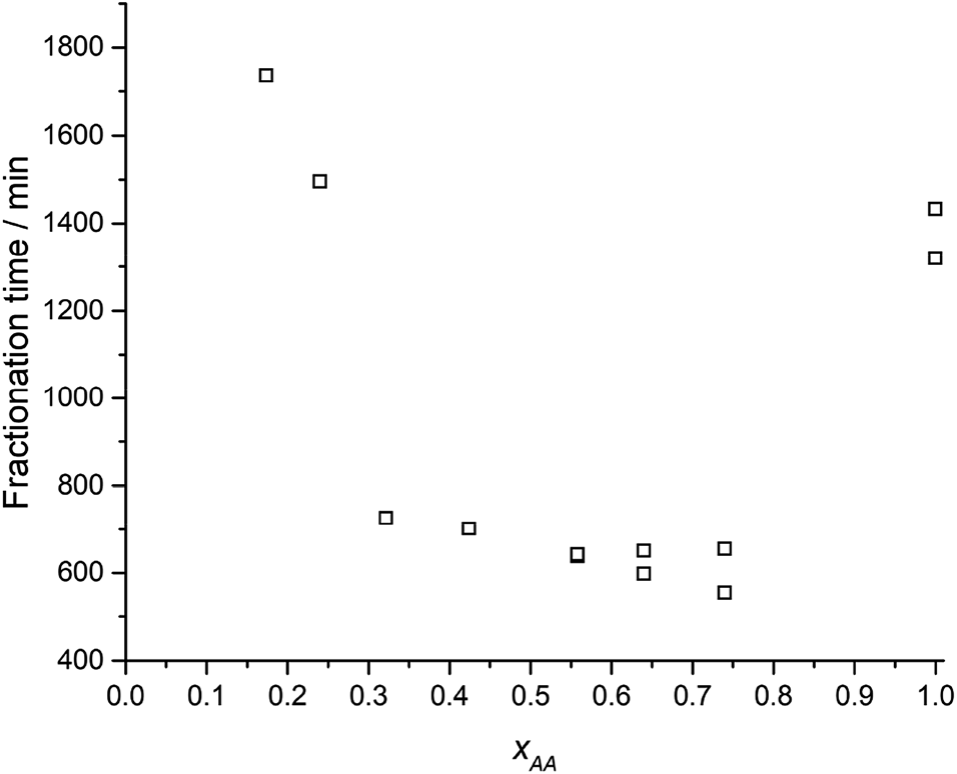
CYS_5_ total fractionation time at 70 °C in various *x*_AA_.

Fig. 2 shows an induction period (horizontal dashed line) followed by a steady state fractionation (solid line) ending at a plateau, giving the total fractionation time (vertical dashed line). Induction periods and steady-state fractionation rates for all acetic acid concentrations can be found in ESI Table S1† and the total fractionation times are given in Fig. 3 as a function of *x*_AA_.

Fig. 3 show that the total fractionation times, under optimal condition at *x*_AA_ = 0.74, is ≈2.5 times smaller than for glacial acetic acid (*x*_AA_ = 1.0) and *x*_AA_ = 0.24. Images (ESI Fig. S8†) shows the CYS_5_ matrix is turned into pulp, except for *x*_AA_ < 0.07. The average fragment size decreased from 0.81 to 0.45 mm (ESI Fig. S9,† left) as *x*_AA_ = 0.32 increased towards *x*_AA_ = 0.74.

### Impact of temperature and l-cystine on the fractionation

**Fig. 4:**
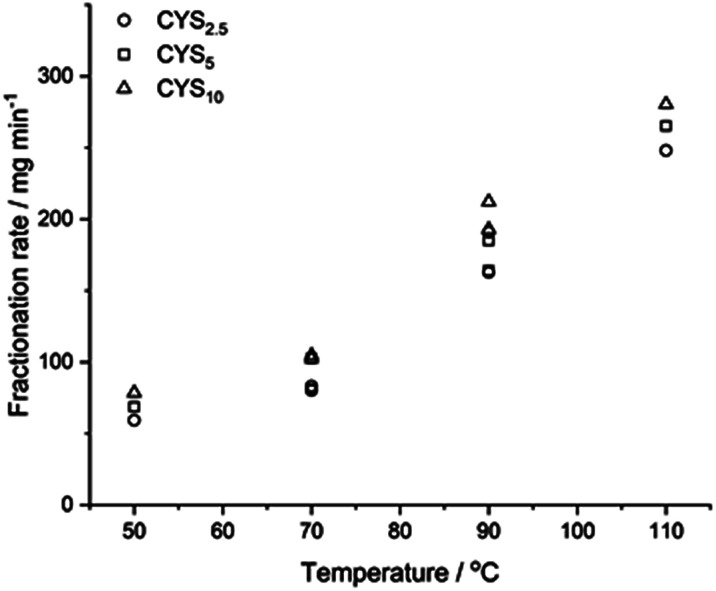
Fractionation of epoxy matrices with different cystine concentrations fractionated in *x*_AA_ = 0.64 as a function of temperature.

Fig. 4 shows the fractionation rate in *x*_AA_ = 0.64 as a function of temperature and l-cystine concentration. The induction period, fractionation time, and total fractionation time is given in ESI Table S2.†

Fig. 4 show that increasing the temperatures from 50 °C to 110 °C increased the fractionation rate from 69 mg min^−1^ to 265 mg min^−1^ (CYS_5_, ≈4 times) where increasing l-cystine concentrations from 2.5% to 10% went from 162 mg min^−1^ to 212 mg min^−1^ (90 °C, ∼1.3 times). Data from CYS_2.5_, CYS_5_, and CYS_10_ can be found in ESI Fig. S10–S12,† and the average particle diameter of the fractionated matrices was 0.6–0.96 mm independent of solvent and l-cystine concentration (ESI Fig. S9†).

### Viscosity profile of modified epoxy resin

The initial viscosities and pot life (time to reach 1.0 Pa s) of the modified epoxy resins are shown in Fig. S14.† The initial viscosity of the epoxy mixtures with different l-cystine concentrations showed a slight ESI increase from 0.18 Pa s to 0.28 Pa s as l-cystine was added. Further, a reduction in pot life from 4.2 h to 2.4 h is seen as l-cystine concentration increase.

### Composite infusion flow rate

Flow front position as a function of time during infusion of VCF is shown in ESI Fig. S15† and 2^nd^ to 4^th^ infusion are shown in ESI Fig. S16–S18,† respectively. It shows linearity between the infused distance squared and infusion time indicating constant flow resistance along the ply during VARTM. Calculated flow rates and resistances based on Darcy's law (eqn (1)) for all infusions are given in Table 2.

**Table 2:** Flow rates (*q*) and resistance (*Kφ*) for 1^st^ to 4^th^ infusion of carbon fabrics. Viscosity 0.19 Pa s and 99 kPa differential pressure

	1^st^	2^nd^	3^rd^	4^th^
*q* [mm^2^ s^−1^]	23.8	620.7	926.9	446.4
*Kφ ×* 10^−5^ [mm^2^]	2.3	59.6	89.0	42.8

**Fig. 5:**
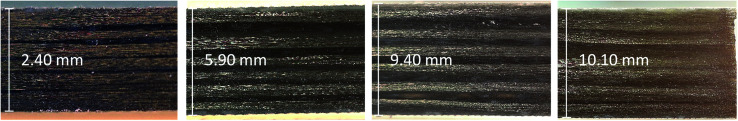
Optical microscope cross-sectional pictures of the composite sandwich structures are shown, and from left to right are 1^st^ to 4^th^ infused composite, respectively.

An apparent increase in infusion speed is observed (Table 2) indicating that the reinfused composites having either a higher infusion permeability or a higher porosity. It was observed during layup that the recycled ply was wavy and non-uniform due to entrapped fragmented epoxy matrix in the fibre bundles. Thus, stacking the recycled ply prior to infusion was challenging and although some compaction took place when vacuum was applied, larger inter ply spacing was found. The 1^st^ to 4^th^ composites' cross-sections are shown in Fig. 5.

Fig. 5 support the decreased packing quality of recycled ply's and show an increase in the inter-ply channels and consequently increased fabric thickness per recycling.

### Volume fractions and flexural moduli of the recycled composites

ESI Fig. S19† shows the TGA results of VCF, recycled fibers, and reinfused composites. A mass reduction of 6 wt% was seen for the VCF at ≈400 °C due to loss of sizing and adsorbed species. The decomposition's onset for the first infusion was 337 °C and for the subsequent infusions 325 °C indicating a slight reduction in thermal stability of the matrix by recycling the carbon fibres. ESI Fig. S19† further showed a decrease in FVF as the plies were recycled. The calculated volume fractions from both burning and pyrolysis are given in Table 3 together with the composites' density and flexural modulus.

**Table 3:** Composite density (*ρ*), ply thickness (*L*_ply_) and total composite thickness (*L*_com_), MVF, FVF, and VVF measured from pyrolysis (N_2_) and burnout (Air), and flexural moduli for the 1^st^ till 4^th^ infusion of the same carbon fabrics

Infusion [#]	1^st^	2^nd^	3^rd^	4^th^
*ρ* [g cm^−3^]	1.43	1.27	1.23	1.18
*L*_ply_ [mm]	0.30	0.61	0.73	0.80
*L*_com_ [mm]	2.40	5.90	9.40	10.10
MVF [%]^a^	50.7 ± 3.9	73.4 ± 0.3	80.9 ± 2.7	82.3 ± 2.8
FVF [%]^a^	46.4 ± 2.5	22.9 ± 0.2	15.8 ± 1.8	11.8 ± 1.8
VVF [%]^a^	2.9 ± 1.4	3.7 ± 0.1	3.3 ± 1.0	3.8 ± 0.1
MVF [%]^b^	66.3	80.3	88.1	88.9
FVF [%]^b^	36.3	18.5	11.2	7.6
VVF [%]^b^	−2.6	1.3	0.8	3.6
Flexural [GPa]	70.8 ± 1.6	14.8 ± 0.7	5.4 ± 0.3	3.9 ± 0.3

a Pyrolysis *via* TGA.

b Burning in an oven ambient atmosphere at 500 °C.

The density and flexural modulus (Table 3 and ESI Fig. S21†) converge towards the clear cast values (1.17 g cm^−3^ and 2.89 GPa)^14^ as the number of recycling increase. Further, the individual ply thickness and the total composite thickness increased as the number of recycling increase and a decrease in FVF when composites are thermal converted with and without oxygen present.

### SEM images of virgin and recycled carbon fibres

**Fig. 6:**
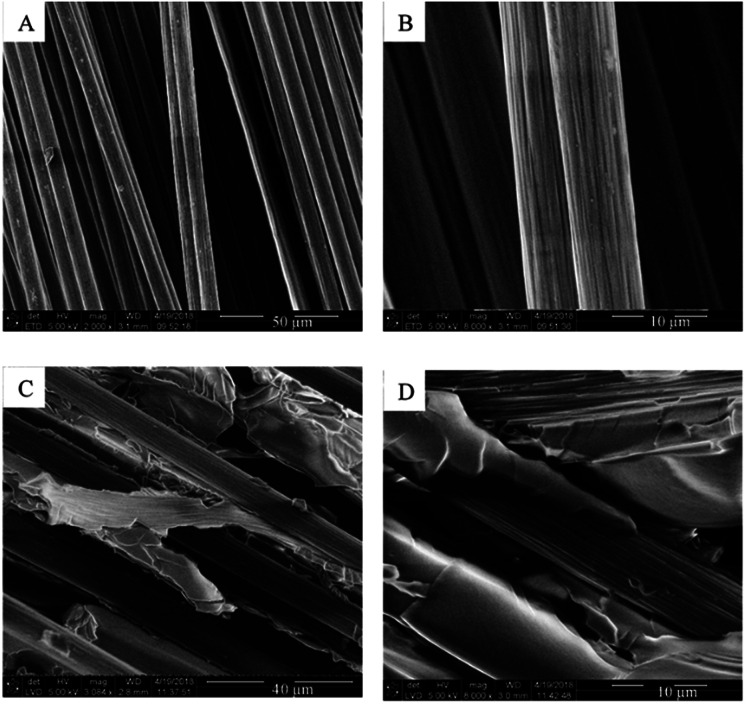
SEM images where (A) and (B) are VCF, (C) and (D) are first time recycled fibres.

SEM images of VCF and recycled carbon fibres are shown in Fig. 6. SEM images of the VCF (Fig. 6A and B) showed clean individual carbon fibres with a textured surface and an average fibre diameter of 7.9 ± 0.5 μm. The textured fibre surface and texture indentation in the epoxy matrix could be observed after the first dismantling (Fig. 6C and D). This demonstrated that the fibres were detached from the matrix and the sharp straight lines on the entrapped epoxy matrix indicated a brittle fracture of the epoxy matrix with clear cuts and no ductile fracture pattern.^16^

### Reshape recycled carbon fabrics

**Fig. 7:**
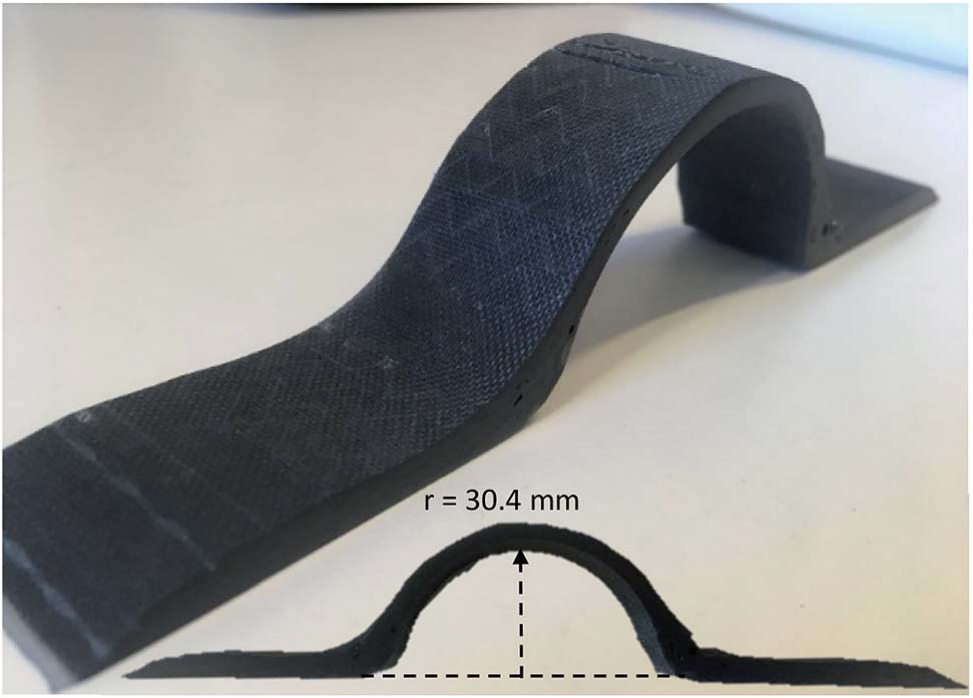
Reshaped carbon composite made from flat recycled carbon fabrics. The inset shows the profile view with curve dimensions.

A flat composite sheet was dismantled and the regained fabrics were reshaped and reinfused into a curved element. Fig. 7 shows the reshaped part.

Fig. 7 demonstrated the potential of reusability by employing l-cystine modified epoxy and a mild recycling method (*x*_AA_ = 0.64, 70 °C, overnight) where long continues carbon fabrics were regained from a composite sheet, which was flexible enough to be reshaped, reinfused and cured into a new shape.

## Discussion

The modified epoxy mixtures' initial viscosities (ESI Fig. S14†) changed from 0.18 Pa s to 0.28 Pa s, which is still well below the 0.5 Pa s limit for infusion resins. Further, up till CYS_10_ no significant change in viscosity is observed compared to the unmodified resin. A reduction (≈25%) in the infusion resins pot life is observed resulting in a reduction in the infusion process window. Both viscosity increase and pot life reduction is ascribed to increased amount and size of resin oligomers after incorporation of l-cystine. Despite these changes, the infusion resins showed homogeneous flow profiles during infusion (ESI Fig S15†).

Full cured epoxy matrices is obtained for all infusion resins as demonstrated by FTIR and DSC. All glass transition temperatures were ≈78 °C and no energy release due to post-curing was observed for any clear casts or composites. This is further supported by the lack of bands from primary and secondary amines (about 3400 cm^−1^), and oxiranes (about 918 cm^−1^) in any FTIR spectra for cured clear cast or composites. Hence, the l-cystine substitution resulted in a balanced infusion resin with expected post-cure properties similar to previous described.^14^

**Fig. 8:**
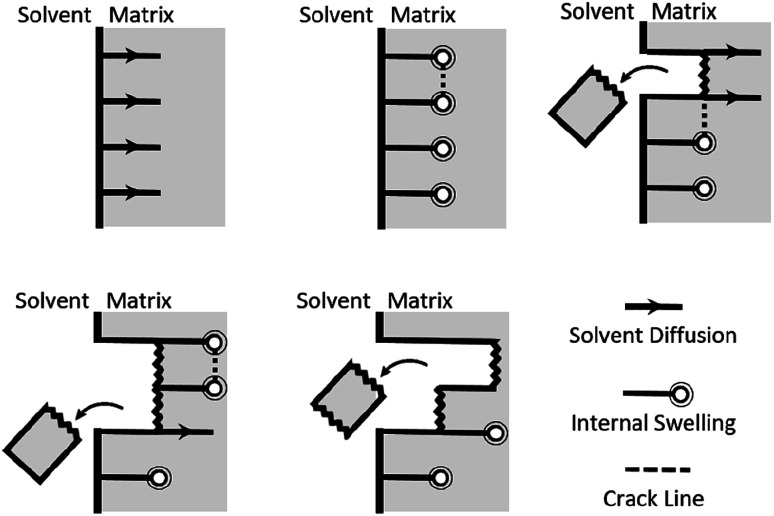
Schematics of the proposed fractionation process.

Optimal composite dismantling conditions were found by fractionation of clear cast samples. l-cystine concentration showed 1.3 times increase in dismantling rate (Fig. 4) and acid dilution showed 2.5 times increase in fractionation rate in the range from *x*_AA_ = 0.32 to *x*_AA_ = 0.74 and an optimum at *x*_AA_ = 0.74 for CYS_5_ (Fig. 3). Further, increasing temperature showed four times increase in the dismantling rate (Fig. 4). It is assumed that the dismantling principal is due to l-cystine introduces increased solvent transport in the matrix and the local matrix swelling breaks off matrix fragments in a brittle fracture process. The hypothesised environmental stress cracking process is illustrated in Fig. 8.

This is supported by the cracking sound and observed, falling fragments during fractionation tests and the fracture patterns observed on the SEM images (Fig. 6C and D). SEM images further show that the epoxy matrix is delaminated from the fibers and the fibers leaves their surface structure imprinted in the epoxy matrix indicating good interfacial contact. The matrix–fiber interface introduces more matrix defects and thereby facilitate solvent transport along fibers. Thus, the introduction of fiber into the epoxy matrices could facilitate composite dismantling indicating that the optimum fractionation condition also applies for the dismantling of the composites.

Carbon fiber reinforced composite panels are successfully recycled (*x*_AA_ = 0.64, 70 °C) and carbon fabrics can be preserved, reinfused, and cured into new panels, as demonstrated three times. SEM images of the dismantled composite (Fig. 6C and D) show that epoxy matrix fragments are encaged in the fibers. Thus, reinfusion results in a reduction of FVF of the cured recycled ply (Table 3 and Fig. 5, left to right). This matrix entrapment also results in less pliable and slightly wavy plies after dismantling and drying, complicating restacking of the recycled plies. Although the ply stack is compressed by the vacuum bag during the infusion, large channels are still present between plies. Resulting in a very high measured infusion rate (Table 2). Despite these inter-ply channels, the reinfusion has infused the entire stack without any entrapped air (Fig. 5 and VVF in Table 3).

There is a linear correlation in determined FVF between TGA and furnace burnout (ESI Fig. S20†). Pyrolysis results in slightly higher FVF due to charring^4*a*^ but both methods clearly show a reduction in FVF of the recycled composites (Table 3, ESI Fig. S19 and S20†). The entrapped epoxy, and as a consequence reduced FVF, reduces the composite strength *i.e.* flexural moduli from 70.8 GPa to 3.9 GPa (Table 3 and ESI Fig. S21†) and in its present form the carbon fiber ply can only be applied in products with lower strength demands. Further studies in minimizing the particle size during dismantling and particle removal from the fiber bundles are to be performed to optimize the recycling and to maintain the mechanical properties.

The l-cystine modified epoxy resin can be applied in infusion production processes with only minor process changes giving it high industrial relevance. The main parameter to consider is the faster viscosity build-up (reduced pot life), which might induce changes in post-infusion resin handling and reduce potential infusion time and distance. Further, the reduced composite strength (FVF) after recycling will challenge many products, but recycled carbon fiber plies might substitute virgin fibers in many low strength demanding products. Thus, this study enables carbon fiber composites to be separated and regain continuous carbon fibers for recycling, which can be reshaped and therefore reprocessed into new products. Further, *x*_AA_ = 0.74 is found to be optimal, but keeping the concentration *x*_AA_ < 0.56 eliminates the fire hazard of the acetic acid solutions^17^ and only sacrificing 10–20% of the dismantling rate. Hence, making the dismantling process safer and applicable to industry. Although the recycled carbon fiber plies are not optimal, it is still far better than incineration or landfill of used composites.

## Conclusions

Carbon fibre reinforced epoxy can be recycled and reshaped after dismantling in 65 vol% acetic acid by addition of only 0.97 wt% of l-cystine to a commercial epoxy system prior to infusion. Infusion, curing, and dismantling cycles are repeated three times. Each recycling shows a reduction in fibre volume fraction as the dismantled fibre bundle's entrapped epoxy matrix resulting in a reduction of the composite's properties. Optimization of the dismantling parameters demonstrate an optimum acetic acid solvent mixture (90 vol%) and increasing temperature (50 to 110 °C) increases the dismantling rate (4 fold) more than an increase in l-cystine concentration (1.3 fold). However, a lower limit of l-cystine, while maintaining recyclability, is not found in this study. To the best of our knowledge, this is among the first reports of recycling a stack of six whole (200 mm *×* 300 mm) plies of continuous long carbon fibers within 24 hours.

## Conflicts of interest

There are no conflicts to declare.

## Supplementary Material

RA-009-C9RA06409E-s001

## References

[cit1] (a) Reinf. Plast., 2017, 61, 213 214, 10.1016/j.repl.2017.06.029

[cit2] Marsh G. (2010). Reinf. Plast..

[cit3] Okajima I., Watanabe K., Sako T. (2012). J. Adv. Res. Phys..

[cit4] Meyer L. O., Schulte K., Grove-Nielsen E. (2009). J. Compos. Mater..

[cit5] Oliveux G., Dandy L. O., Leeke G. A. (2015). Prog. Mater. Sci..

[cit6] Jin K., Li L., Torkelson J. M. (2016). Adv. Mater..

[cit7] Yu K., Shi Q., Dunn M. L., Wang T., Qi H. J. (2016). Adv. Funct. Mater..

[cit8] Zhang Y., Ying H., Hart K. R., Wu Y., Hsu A. J., Coppola A. M., Kim T. A., Yang K., Sottos N. R., White S. R., Cheng J. (2016). Adv. Mater..

[cit9] Hashimoto T., Meiji H., Urushisaki M., Sakaguchi T., Kawabe K., Tsuchida C., Kondo K. (2012). J. Polym. Sci., Polym. Chem..

[cit10] Wang L., Li H., Wong C. P. (2000). J. Polym. Sci., Polym. Chem..

[cit11] Ruiz de Luzuriaga A., Martin R., Markaide N., Rekondo A., Cabanero G., Rodriguez J., Odriozola I. (2016). Mater. Horiz..

[cit12] Ma S., Webster D. C., Jabeen F. (2016). Macromolecules.

[cit13] You S., Ma S., Dai J., Jia Z., Liu X., Zhu J. (2017). ACS Sustainable Chem. Eng..

[cit14] Henriksen M. L., Ravnsbæk J. B., Bjerring M., Vosegaard T., Daasbjerg K., Hinge M. (2017). ChemSusChem.

[cit15] Socrates G. , Infrared and Raman Characteristic Group Frequencies, 3rd edn, Wiley, 1994

[cit16] Davim J. P. and Charitidis C. A., Nanocomposites: Materials, Manufacturing and Engineering, De Gruyter, 2013

[cit17] U. S. Pipeline and Hazardous Materials Safety Administration , Emergency Response Guidebook 2016, U.S. Dept. of Transportation, Pipeline and Hazardous Materials Safety Administration, 2016

[cit18] Henriksen M. L. , Friis J. E., Rydder S. and Hinge M., Open Science Framework, 2017, 10.17605/OSF.IO/R5GW4

[cit19] Sirtautas J., Pickett A. K., George A. (2015). Appl. Compos. Mater..

