# Supplementary material for: Recyclable carbon fibre composites enabled by cystine containing epoxy matrices
Source: RSC Adv. 2019 Oct 2;9(54):31378–85. doi: 10.1039/c9ra06409e (PMC9072556; doi:10.1039/c9ra06409e)
Supplement: RA-009-C9RA06409E-s001 [file RA-009-C9RA06409E-s001.pdf]

Electronic Supplementary Material (ESI) for RSC Advances.
This journal is © The Royal Society of Chemistry 2019

Supporting Information

Recyclable Carbon Fibre Composites Enabled by Cystine Containing Epoxy Matrices

*Dr. Martin L. Henriksen, Dr. Jakob E. Friis, Astrid Voss, and Assoc. Prof. Mogens Hinge**

Plastic and Polymer Engineering, Department of Engineering, Aarhus University, Aabogade 40, 8200 Aarhus N, Denmark

E-mail: hinge@eng.au.dk

Modified epoxy matrices

The amount of hardener and additive needed is calculated based on Eq. S1.

$$n_{Add} = X\frac{\left(n_{ER}\,\#_{AS_{ER}}\right)}{\#_{AS_{Add}}};\; n_H = (1 - X)\frac{\left(n_{ER}\,\#_{AS_{ER}}\right)}{\#_{ASH}} \qquad \text{(Eq. S1)}$$

Where *ER*, *H* and *Add* denotes Epoxy Resin, Hardener and Additive. *#AS* is the number of active sites, *n* is the number of moles, and *X* is the additive mole percentage.

FTIR

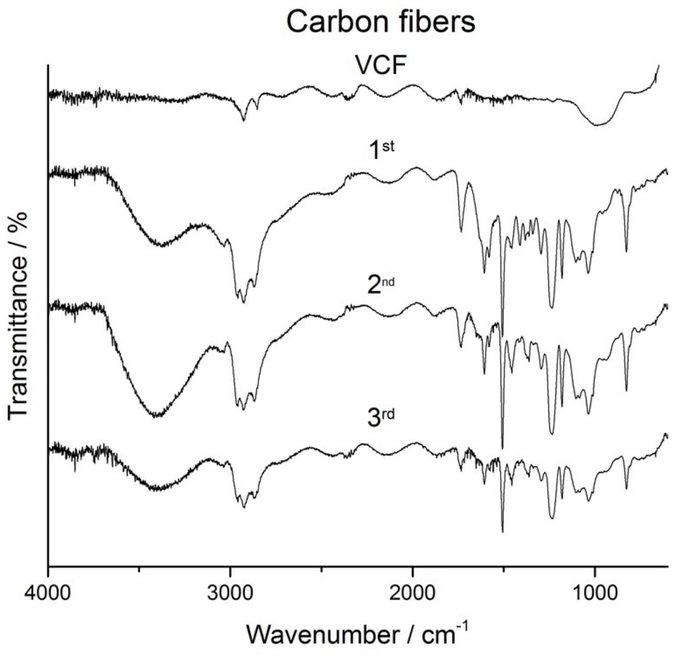

Fig. S1 FTIR of the virgin and recycled carbon fibres

DSC

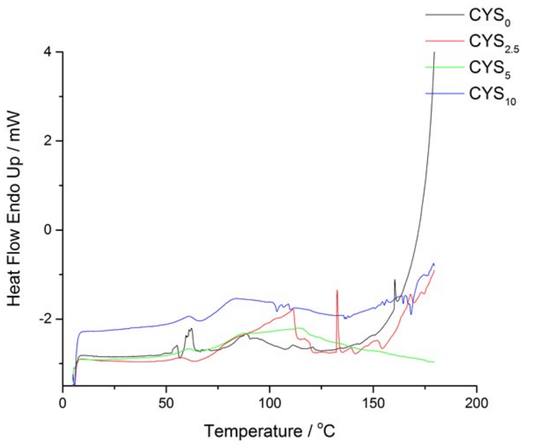

Fig. S2 1st Heat Run Epoxy Matrices

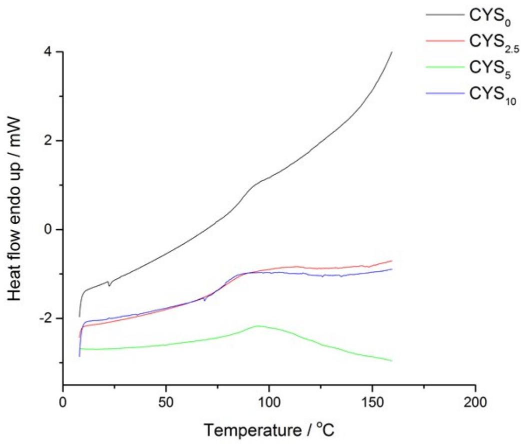

Fig. S3 2nd Heat Run Epoxy Matrices

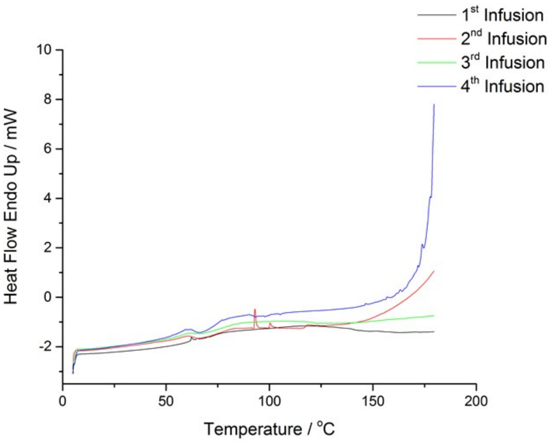

Fig. S4 1st Heat Run Composite

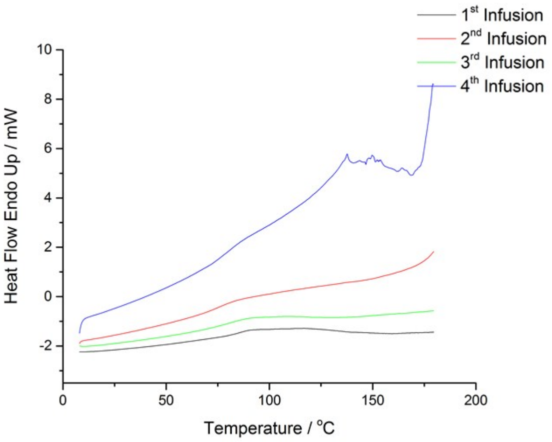

Fig. S5 2nd Heat Run Composite

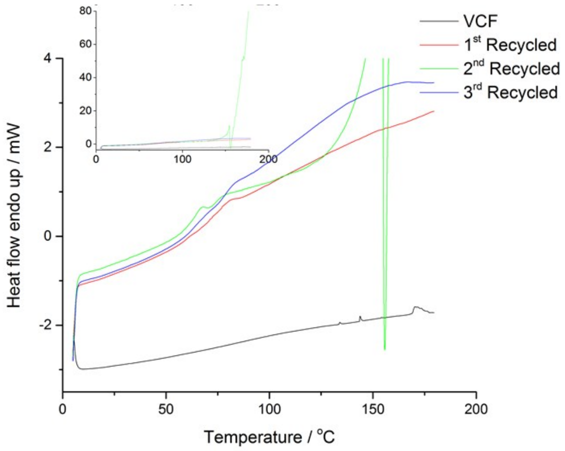

Fig. S6 1st Heat Run Fibres

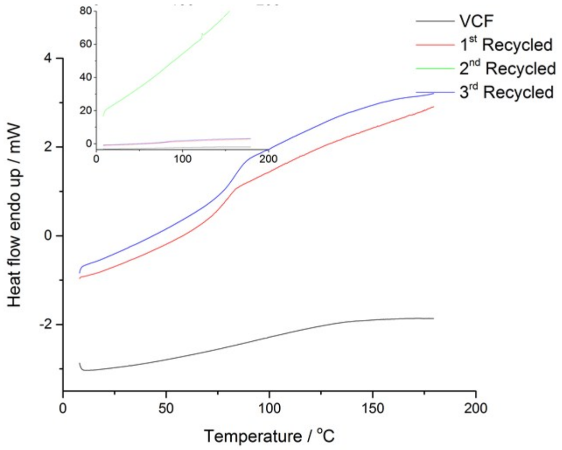

Fig. S7 2nd Heat Run Fibres

Optimal dismantling rates

Table S1 Various mole fraction acetic acid fractionation times for CYS5.

x_{AA}	0.17	0.24	0.32	0.42	0.56	0.56	0.64	0.64	0.74	0.74	1.00	1.00
Induction period [min$^{1/2}$]	8.9	8.2	10.6	5.0	6.1	4.8	3.5	3.6	6.3	2.2	8.0	6.4
Fractionation time [min$^{1/2}$]	32.7	30.4	16.3	21.5	19.2	20.5	20.9	21.9	17.3	23.4	29.8	29.9
Total fractionation time [min$^{1/2}$]	41.7	38.7	26.9	26.5	25.3	25.3	24.5	25.5	23.5	25.6	37.8	36.3

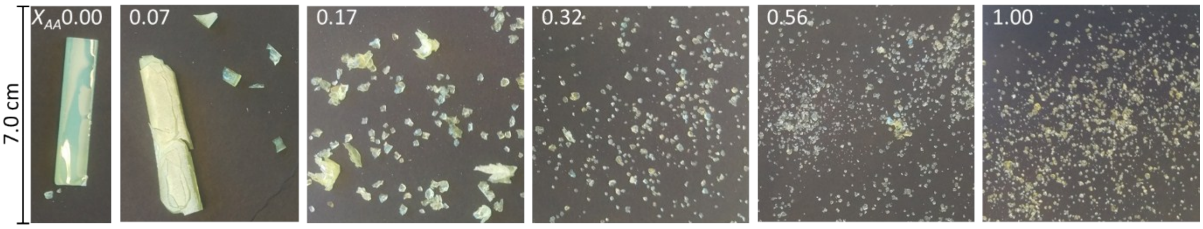

Fig. S8 CYS$_5$ test specimens after submersion in 0.00, 0.07, 0.17, 0.32, 0.56, and 1.00 mole fraction acetic acid at 70 °C, respectably.

The effect of acetic acid can be seen in Fig. S8 for CYS5 submerged at 70 °C. 0.00 and 0.07 acetic acid mole fraction does not show an effect, whereas higher mole fractions shows a higher degree of fractionation of the epoxy matrix into a plethora of fine lumps.

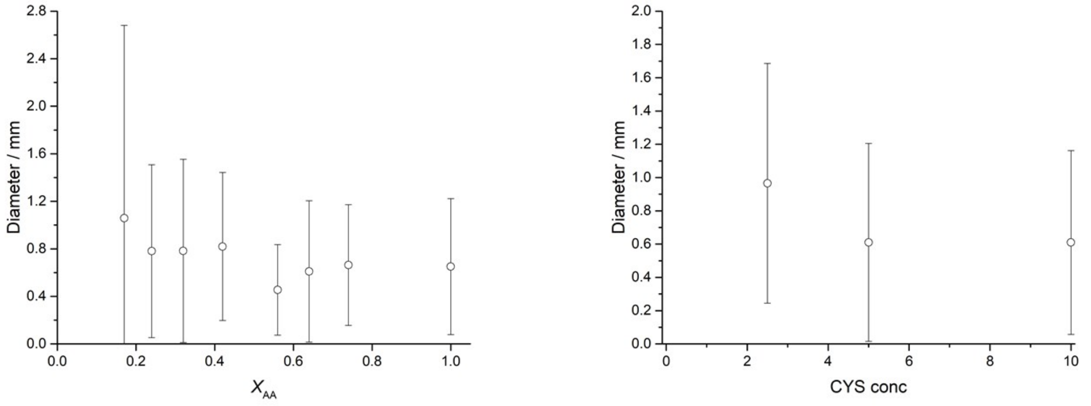

Fig. S9 particle sizes measured with picture analysis. To the left is the particle sizes for various acetic acid concentrations at 70 °C and CYS$_5$. To the right is the particle sizes for various cystine additive at 70 °C and 0.64 mole fraction acetic acid.

The pulp was regained by sedimentation, dried and analysed by picture analysis. For each measurements, 2000-6000 particles have been counted and the diameters together with the standard deviations are shown in Fig. S9.

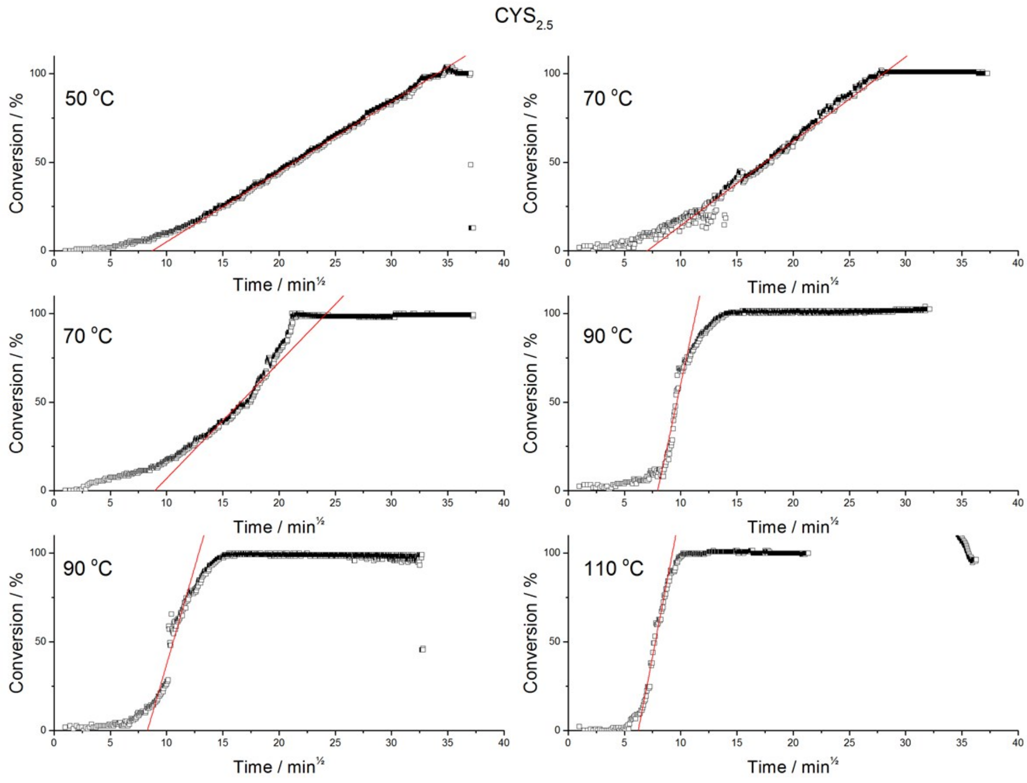

Fig. S10 Raw data plotted at square root time following the fractionation rate for $CYS_{2.5}$ in x_{AA} = 0.64 at elevated temperatures.

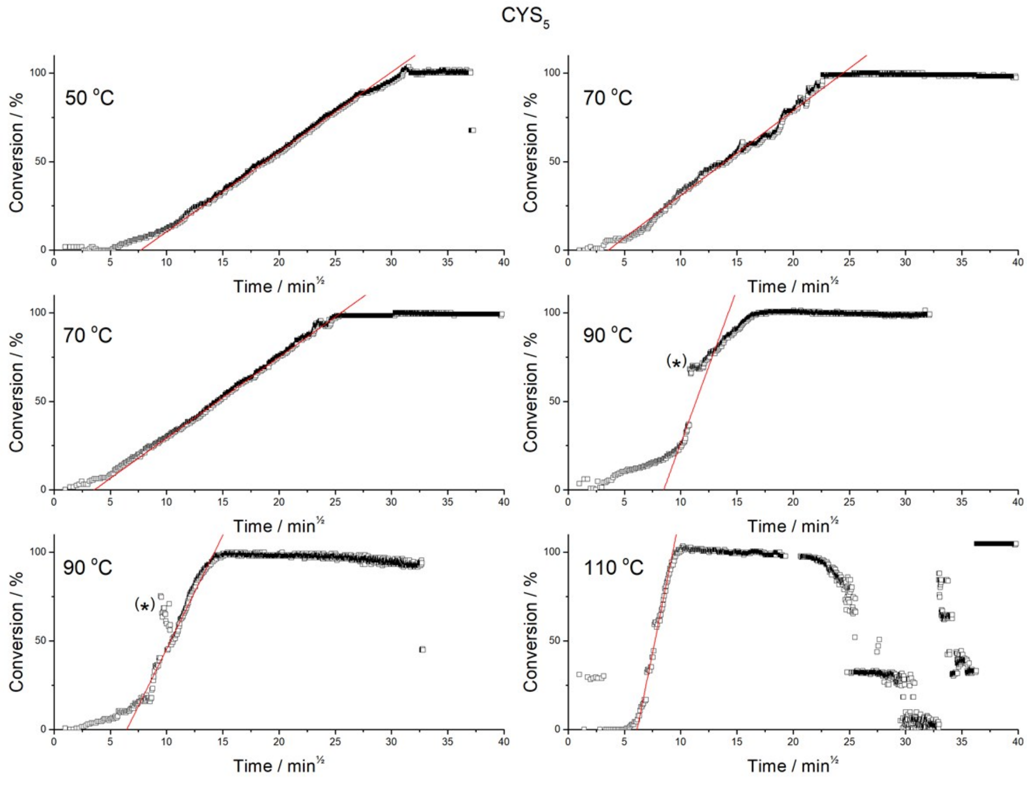

Fig. S11 Raw data plotted at square root time following the fractionation rate for CYS_5 in x_{AA} = 0.64 at elevated temperatures. Stars indicates regions that are removed when fitting the fractionation time.

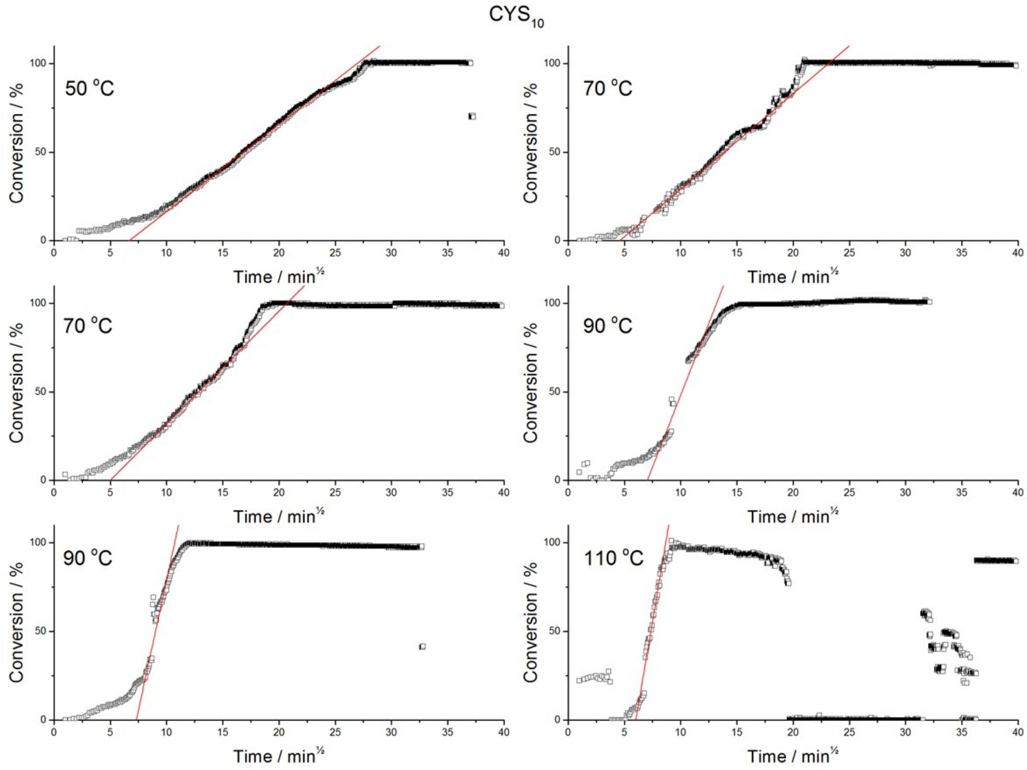

Fig. S12 Raw data plotted at square root time following the fractionation rate for CYS_{10} in x_{AA} = 0.64 at elevated temperatures.

Table S2 Temperature and CYS concentration fractionation times in minutes^{½}.

	Induction period [min^{½}]			Fractionation time [min^{½}]			Total fractionation time [min^{½}]		
Temp [°C]	$CYS_{2.5}$	CYS_5	CYS_{10}	$CYS_{2.5}$	CYS_5	CYS_{10}	$CYS_{2.5}$	CYS_5	CYS_{10}
50	8.7	7.7	6.7	25.3	22.2	20.3	34.1	29.9	26.9
70	7.0	3.5	4.6	21.0	20.9	18.6	28.0	24.5	23.1
70	9.0	3.6	5.0	15.3	21.9	15.7	24.2	25.5	20.7
90	8.0	8.5	7.0	3.4	5.7	6.2	11.4	14.2	13.2
90	8.3	6.5	7.3	4.6	7.8	3.4	12.9	14.3	10.7
110	6.2	6.1	6.0	3.0	3.2	2.7	9.3	9.3	8.7

Rheology

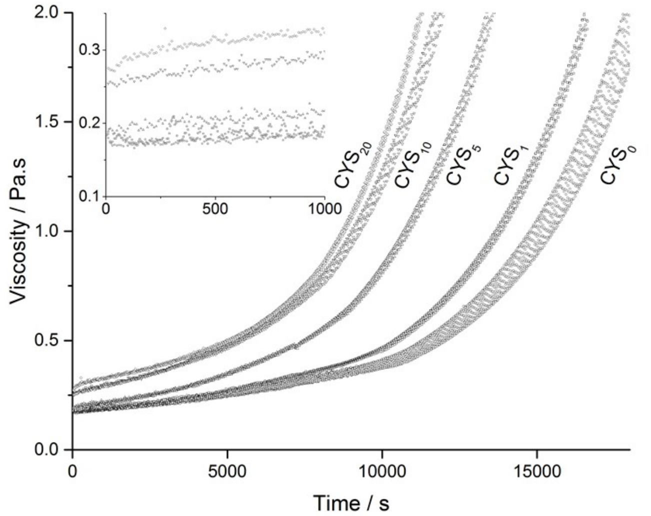

Fig. S13 the viscosity of the standard and modified epoxy mixtures are done at 30 °C and shows a slight increase for the initial viscosity together with a higher curing rate for the modified systems.

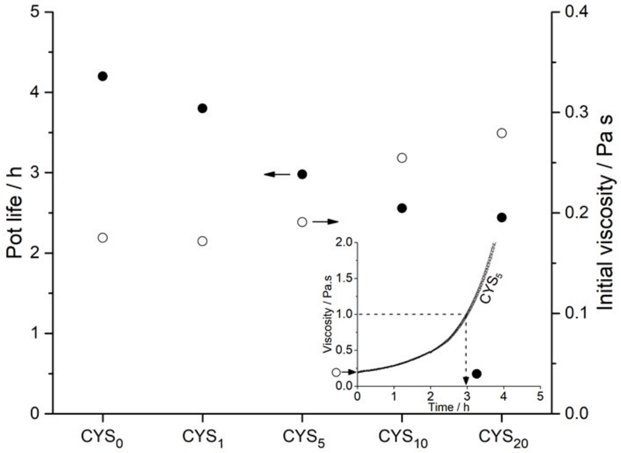

Fig. S14 Pot life (full circles) and the initial viscosity (open circles) as a function of L-cystine concentration determined at 30 °C by shear rheometry. Insert shows the viscosity profile as a function of time for CYS5 and determination of initial viscosity and pot life.

Composite infusion flow rates

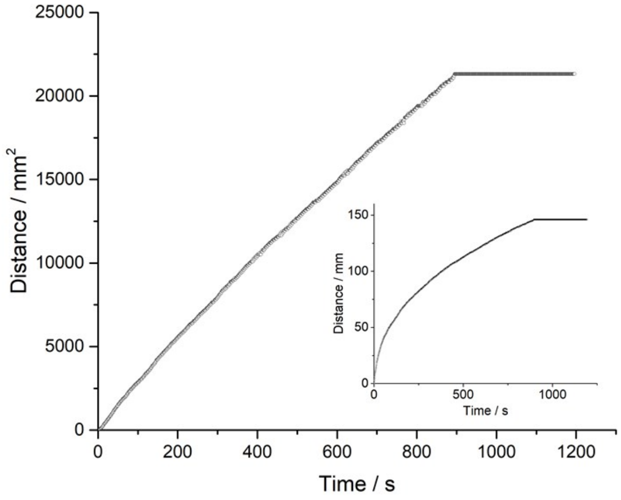

Fig. S15 The VCF infusion's flow front as a function time versus squared distance. The insert shows the raw data of the same flow front as time versus distance.

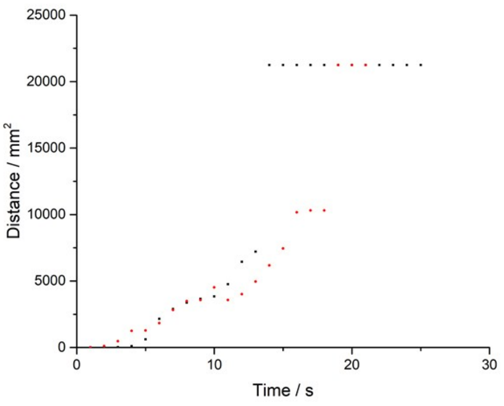

Fig. S16 The flow front's position for the 2nd infusion measured at two individual segments.

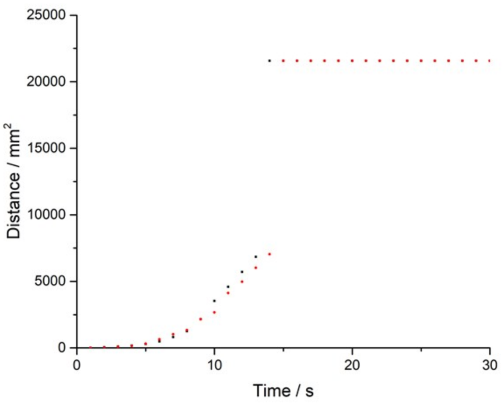

Fig. S17 The flow front's position for the 3rd Infusion measured at two individual segments.

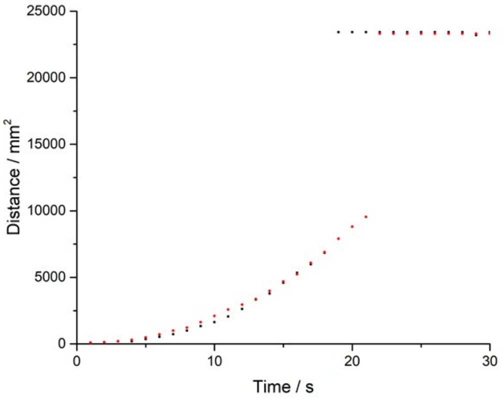

Fig. S18 The flow front's position for the 4th Infusion measured at two individual segments.

TGA

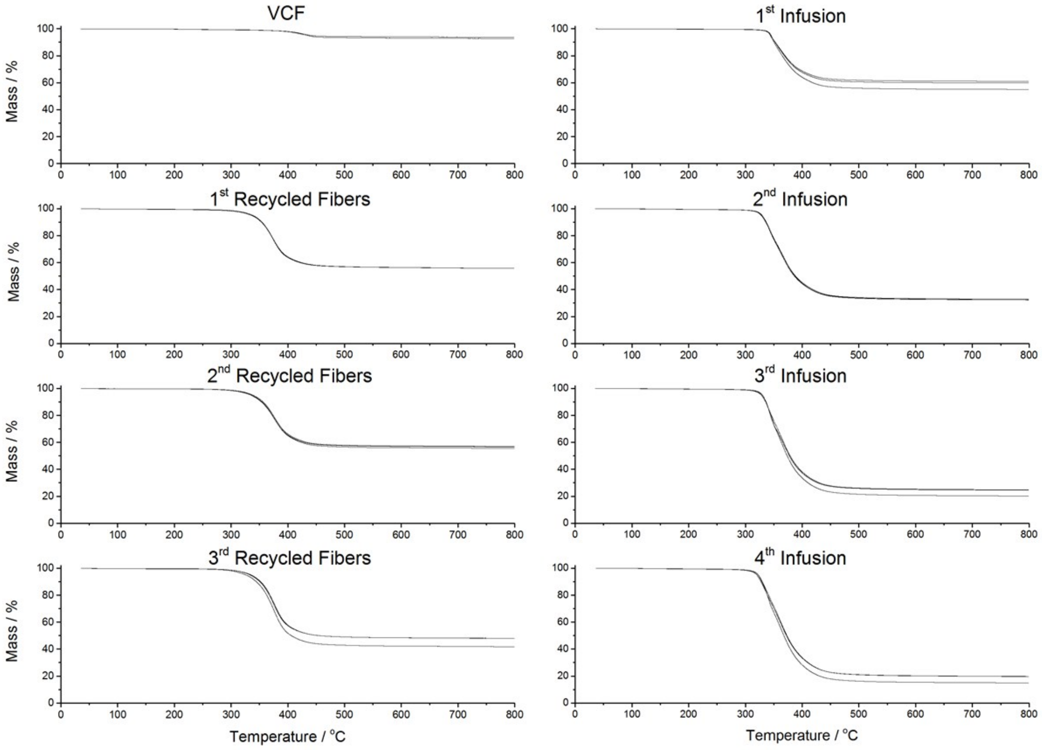

Fig. S19 TGA raw data for VCF, composites and regained fabrics done in triplicates.

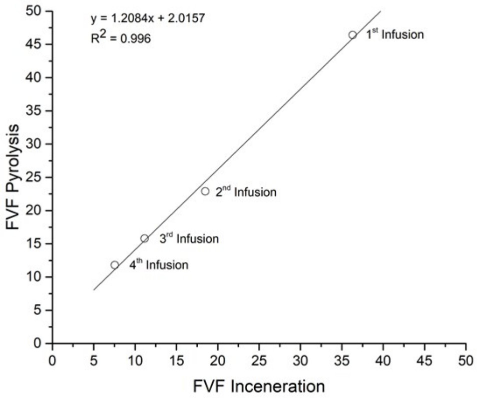

Fig. S20 Correlation plot between FVF of composites measured via burning in inert atmosphere and with oxygen. There is a good correlation between both methods and the difference is found in the incineration process, where TGA uses N_2 and the oven uses natural air.

Three point bending

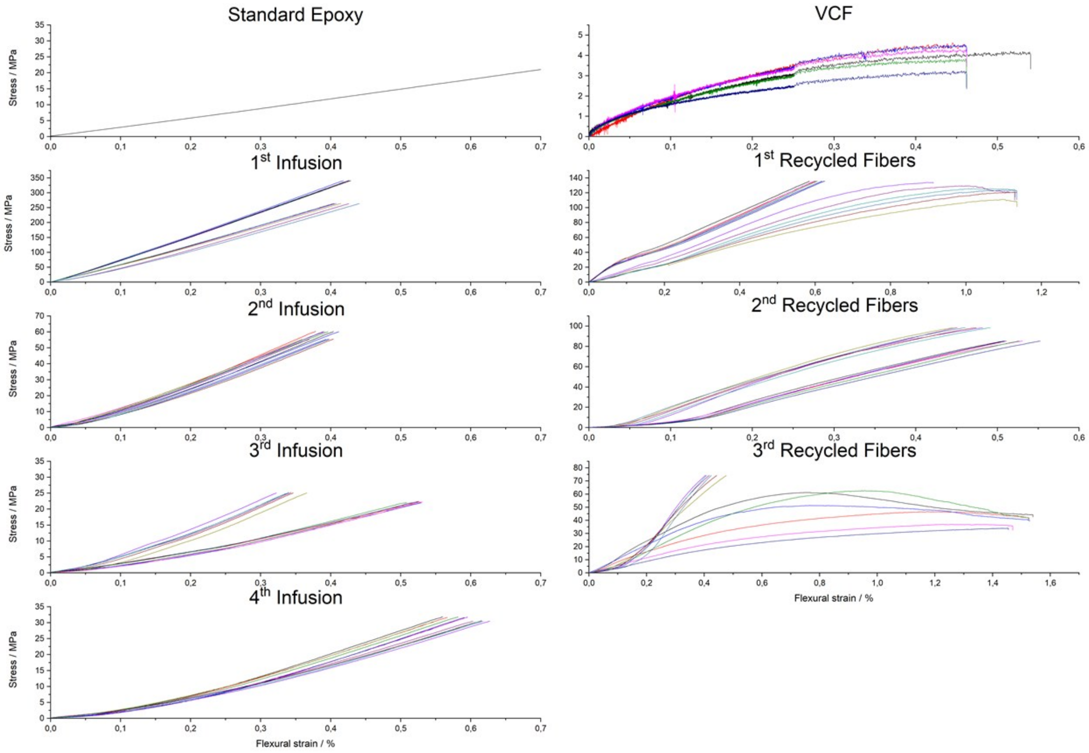

Fig. S21 Three point bending of fibres and composites. Test coupons are measured at various spots in order to determine the flexural properties. The material become softer as the epoxy fraction increase in the recycled fibres and the recycled composites per cycle.

Recycled carbon fabrics residual epoxy and stiffness

Table S3 Virgin and recycled fibres flexural properties and the mass after pyrolysis.

	Standard epoxy	VCF	1st Recycled Fibres	2nd Recycled Fibres	3rd Recycled Fibres
Mass left [%][a]	-	93.98±1.31[a]	55.76±0.04[a]	56.43±0.7[a]	45.91±3.61[a]
Flexural modulus [GPa]	2.89	0.98±0.19	15.36±1.72	22.46±1.2	11.66±2.34

[a] pyrolysis via TGA

Macro for ImageJ made for sedimentation height analysis.

Copy the code below into a ".txt" document and open it via ImageJ:

Plugins→Macros→Run…→Click on ".txt" file

//**File handling**.

bilstart = 1; //Indicates first picture

bilslut = 1389; //Indicates the number of pictures to be analyses (picture number_end – picture number_start)

base = "C:\\Users\\...\\picturefolder\\picturename"; // remember to use double "\\" for directory, when using ImageJ macro

picturetype = ".bmp" // .JPEG .BMP .png etc. (remember the "." period)

//**Indicates the area to analyse. The values are added manually**.

startx = 400; // starting x position (remember (0,0) is in top left corner)

starty = 100; // starting y position

deltax = 150; // The width of the area om x-direction

deltay = 280; // The width of the area in y-direction

endx = startx+deltax // end x position

rot = 0; //Insert rotation angle

ymindste = 0; //Insert lowest pixel in y-direction to analyse

black = 85; // the black tolerance: 0 is all black, 225 is all white. It starts at 0 and define to where.

print("Working on converting "+bilslut+" images"); // writing information to the user

setBatchMode(true); //Avoids pictures to be shown

```
run("Clear Results"); //Removes any old data in the result column
Teller=0;

for(j=bilstart; j<=(bilslut+bilstart); j++) {
showProgress(j, bilslut);

navn = j; //Creates file name for files named 1,2,3...

//if (j<10) {navn = "000"+j; } //Creates file name with leading zero for files named 0001,
0002, 0003...
//if (j>9) {navn = "00"+j; }
//if (j>99) {navn = "0"+j; }
//if (j>999) {navn = ""+j; }

sti = base+navn+picturetype; //Compiling the file path

open(sti); //Opens this file
input = getImageID(); //Saves name of the picture

//Picture transformation prior to picture analyses.
run("8-bit"); //Creates 8-bit picture to reduce the amount of computation
run("Rotate... ", "angle=rot grid=1 interpolation=Bilinear"); //Rotates the picture
makeRectangle(startx, starty, deltax, deltay); //Retrieves area of interest
run("Crop"); //Crops the picture to reduce the amount of computation
setAutoThreshold("Default B&W"); //Creates threshold in B&W
setThreshold(0, black); //Indicates the threshold boundaries
```

run("Convert to Mask"); //Makes B&W (binary)

//**Algorithm to picture analysis**.

k=1; //Indicates the result column

maks=0;

sted=0;

for(y=0; y<=deltay-1; y++) { //Runs downwards the picture in the y-direction

resul=0; //Resets the outcome variable

for(x=0; x<=deltax-1; x++) { //Runs across the picture in the x-direction

resul = resul+ getPixel(x,y); //Retrieves and adds the colour for pixel (x,y) to the result column

}; //Done adding values

if (y>ymindste){if(resul>maks){maks=resul;sted=y;};}; //When far enough down the picture the highest values are noted in maks and it is noted where in the picture it happens

}; //Done calculating on the rows in the y-direction

setResult(k, Teller, sted); //Gives result format with results in k, j

setResult(k+1, Teller, navn); //Gives result format with results in k, j

selectImage(input); //Selects the picture again

//**Adding a line for visual inspection of calculation**.

setTool("line"); //Choose line tool

setColor(255,40,0); //Give it a colour you like (R,G,B)

setForegroundColor(255, 40, 0); //Give it a colour you like (R,G,B)

makeLine(0, sted, deltax, sted,2); //From where to where – there is defined variables for all coordinates

//makeLine(x1, y1, x2, y2, lineWidth) //For thicker line if you feel like

```
run("Draw", "slice"); //Line is drawn
gembillede = base+"_"+Teller+".bmp"; //The path for the marked image
saveAs("BMP", gembillede); //The image is saved now

run("Close"); //Closes the picture
Teller=Teller+1;
}; //Next picture is analysed

//Saving results.
gemnavn = base+"resultat.txt"; //Concatenating the result filename
updateResults(); //Updating the result table
saveAs("text",gemnavn); //Saving the data

setBatchMode(false); //Show images again
print("DONE!"); //Let the user know it is done
```

Macro for ImageJ made for flow front analysis

Copy the code below into a ".txt" document and open it via ImageJ:

Plugins→Macros→Run…→Click on ".txt" file

File handling.

bilstart = 1; //Indicates first picture

bilslut = 1224; //Indicates the number of pictures to be analyses (picture number_end – picture number_start)

base = "C:\\\\Users\\\\...\\\\picturefolder\\\\picturename"; // remember to use double "\\\\" for directory, when using ImageJ macro

picturetype = ".bmp" // .JPEG .BMP .png etc. (remember the dot)

//**Indicates the area to analyze. The values are added manually**.

startx = 150; // starting x position (remember (0,0) is in top left corner)

starty = 60; // starting y position

deltax = 50; // The width of the area om x-direction

deltay = 360; // The width of the area in y-direction

endx = startx+deltax // end x position

rot = 0; //Insert rotation angle

ymindste = 0; //Insert lowest pixel in y-direction to analyse

black = 140; // the black tolerance: 0 is all black, 225 is all white. It starts at 0 and define to where.

print("Working on converting "+bilslut+" images"); // writing information to the user

setBatchMode(true); //Avoids pictures to be shown

```
run("Clear Results"); //Removes any old data in the result column
Teller=0;

for(j=bilstart; j<=(bilslut+bilstart); j++) {
showProgress(j, bilslut);

navn = j; //Creates file name for files named 1,2,3...

//if (j<10) {navn = "000"+j; } //Creates file name with leading zero for files named 0001,
0002, 0003...
//if (j>9) {navn = "00"+j; }
//if (j>99) {navn = "0"+j; }
//if (j>999) {navn = ""+j; }

sti = base+navn+picturetype;

open(sti); //Opens this file
input = getImageID(); //Saves name of the picture

//Picture transformation prior to picture analyse.
run("8-bit"); //Creates 8-bit picture to reduce the amount of computation
run("Rotate... ", "angle=rot grid=1 interpolation=Bilinear"); //Rotates the picture
makeRectangle(startx, starty, deltax, deltay); //Retrieves area of interest
run("Crop"); //Crops the picture to reduce the amount of computation
setAutoThreshold("Default B&W"); //Creates threshold in B&W
setThreshold(0, black); //Indicates the threshold boundaries
```

run("Convert to Mask"); //Makes B&W (binary)

//**Algorithm to picture analysis**.

k=1; //Indicates the result column

maks=0;

sted=0;

for(y=0; y<=deltay-1; y++) { //Runs downwards the picture in the y-direction

resul=0; //Resets the outcome variable

for(x=0; x<=deltax-1; x++) { //Runs across the picture in the x-direction

resul = resul+ getPixel(x,y); //Retrieves and adds the colour for pixel (x,y) to the result column

}; //Done adding values

if (y>ymindste){if(resul>maks){maks=resul;sted=y;};}; //When far enough down the picture the highest values are noted in maks and it is noted where in the picture it happens

}; //Done calculating on the rows in the y-direction

setResult(k, Teller, sted); //Gives result format with results in k, j

setResult(k+1, Teller, navn); //Gives result format with results in k, j

selectImage(input); //Selects the picture again

//**Adding a line for visual inspection of calculation**.

setTool("line"); //Choose line tool

setColor(255,40,0); // give it a colour you like (R,G,B)

setForegroundColor(255, 40, 0); //Give it a colour you like (R,G,B)

makeLine(0, sted, deltax, sted,2); //From where to where – there is defined variables for all coordinates

//makeLine(x1, y1, x2, y2, lineWidth) //For thicker line if you feel like

```
run("Draw", "slice"); //Line is drawn
gembillede = base+"_"+Teller+".bmp"; //The parth for the marked image
saveAs("BMP", gembillede); //The image is saved now

run("Close"); //Closes the picture
Teller=Teller+1;
}; //Next picture is analysed

//Saving results.
gemnavn = base+"resultat.txt"; //concatenating the result filename
updateResults(); //updating the result table
saveAs("text",gemnavn); //saving the data

setBatchMode(false); //show images again
print("DONE!"); //let the user know it is done
```
